# A Wearable Button Antenna Sensor for Dual-Mode Wireless Information and Power Transfer †

**DOI:** 10.3390/s21175678

**Published:** 2021-08-24

**Authors:** Jiahao Zhang, Jin Meng, Wei Li, Sen Yan, Guy A. E. Vandenbosch

**Affiliations:** 1 National Key Laboratory of Science and Technology on Vessel Integrated Power System, Wuhan 430033, China; mengjinemc@163.com (J.M.); rf_liwei@163.com (W.L.); 2 School of Information and Communications Engineering, Xi’an Jiaotong University, Xi’an 710049, China; sen.yan@xjtu.edu.cn; 3 WAVECORE Research Division, Department of Electrical Engineering, KU Leuven, 3001 Leuven, Belgium; guy.vandenbosch@esat.kuleuven.be

**Keywords:** wearable sensors, button antennas, wireless power transfer, dual-band antenna, circularly polarized antenna, artificial magnetic conductor

## Abstract

A novel wearable button antenna sensor is proposed for the concept of simultaneous wireless information and power transfer (SWIPT). This integrates two working modes for the transfer of power and information, respectively, and optimizes transfer efficiency. An omni-directional radiation pattern is achieved in the 3.5 GHz World Interoperability for Microwave Access (WiMAX) band to support on-body wireless communications, while a circularly polarized broadside radiation pattern is obtained in the 5 GHz wireless local area networks (WLAN) band to harvest power. The measured −10 dB return loss bandwidths are 4.0% (3.47–3.61 GHz) in the lower band, and 25.0% (4.51–5.80 GHz) in the higher band, respectively. An artificial magnetic conductor (AMC) structure with wideband characteristics is applied to obtain a low-profile design and to increase the stability of the antenna sensor. A high radiation efficiency of over 80% in the whole working band is observed. The specific absorption rate (SAR) of the proposed antenna sensor is below 0.509 W/kg at 3.55 GHz, and below 0.0532 W/kg at 5.5 GHz, respectively, which is much lower than the European standard threshold of 2 W/kg. All these characteristics make the designed antenna sensor suitable for on-body information transmission and off-body energy harvesting. The antenna sensor has been prototyped. Simulations and measurements agree well, proving the validity of the new concept.

## 1. Introduction

Wireless body area networks (WBAN) hold great promise for their rich potential in diverse areas, e.g., medical monitoring, physical training, navigation, wearable computing, etc. [1,2,3]. Therefore, WBAN has attracted considerable research interest and industrial investment over the past decade [4]. As one of the most important aspects of WBAN, wearable antenna sensors have received increasing research attention and are in urgent demand for many wearable applications [5,6,7]. They are able to send wireless information and power transmission from an on-body sensor node to an off-body (information or power) node, or to another on-body sensor node [8]. Modern wireless systems usually have to combine multiple standards and protocols [9]. This means that wearable antenna sensors with multiple functions are urgently required.

The concept of simultaneous wireless information and power transfer (SWIPT) has emerged as a potential idea to realize various future applications [10,11], including WBAN. Some designs have combined near-field power transmission and far-field communication for WBAN applications [12]. This method is suitable for implanted devices since the power transmission distance is limited in this scenario. The far-field power and information transmission, which is enabled by wearable antenna sensors, is also desired in many other scenarios. The specific design of the wearable antenna sensors for SWIPT is quite different from conventional designs. User comfort and a good radiation performance for both SWIPT functions are crucial parameters.

For on-body information transmission, wearable antenna sensors with omni-directional radiation are consistently preferred [13,14], since they can cover a larger wireless communication area to compensate for human body movement.

Wireless power transfer (harvesting) for wearable applications has received recent attention [15,16]. Wearable antennas are used to receive or harvest wireless power to supply on-body sensors, which avoids the need for batteries so that the sensors can be worn for longer periods of time. Antennas with broadside radiation patterns are commonly used for wireless power transfer, since in wearable applications, only the energy from the forward direction can be utilized, as the backward direction is shielded by the human body. Additionally, circularly polarized wearable antennas are preferred over linearly polarized antennas for wireless power transmission applications due to their robustness with respect to orientation. Note that in realistic wearable circumstances, a polarization mismatch always exists for linearly polarized transmitting and receiving antennas, due to the movements and possible postures of the human body. In summary, on-body antennas with broadside radiation pattern and circular polarization are superior for wearable energy harvesting.

Information transfer and energy harvesting with wearable antennas have been investigated separately [17,18,19]. However, the designs, which integrate these two functions in a single antenna sensor, are limited. Button antennas with dual-band operation were studied for on-body applications [14,20,21]. However, they are not ideal for wireless power transfer because circular polarization is not achieved, which in turn makes these designs unrobust when polarization mismatch occurs due to human body movements. On-body antennas for wireless power transfer (harvesting) were presented in [15,22,23], however, only with a single operating band and a linearly polarized radiation pattern, which limits their applications. A dual-band antenna design was proposed in [24]. However, circular polarization was not achieved. Circularly polarized wearable antennas were designed in [18,25]. The design in [18] is dedicated to wireless power transmission. However, the antenna efficiency is relatively low, which decreases the power transmission efficiency. The bandwidth is narrow and in only one operating band in [25], which limits its applications. A dual-band button antenna with both linear and circular polarization was proposed in [26]. However, the button structure is relatively complex since many rivets were used.

Among different wearable antenna topologies, button antennas show clear advantages, since they are conformally designed with rigid buttons, which usually guarantees higher radiation efficiencies than pure textile wearable antennas. This feature is quite important for wireless power transfer or harvesting.

In this paper, a novel dual-mode wireless information and power transfer implementation is developed, optimizing the efficiency for both the energy and the information transfer. It involves two frequencies and is enabled by the proposed dual-mode wearable antenna sensor, whose topology was inspired by the concept of the artificial magnetic conductor (AMC) metamaterial. The antenna sensor is based on a crossed dipole topology, which can generate a broadside radiation pattern with circular polarization. Furthermore, a monopole-like resonant mode with omni-directional radiation pattern is obtained in the lower operating band, which clearly distinguishes the designed antenna from other crossed dipole antennas [27,28]. This monopole-like radiation pattern is used for on-body information transfer in the 3.5 GHz World Interoperability for Microwave Access (WiMAX) band. The broadside radiation pattern with circularly polarization is utilized for wireless power transfer in the 5 GHz wireless local area networks (WLAN) band. In order to mimic a realistic situation, an AMC with wideband characteristics is applied to obtain a low-profile design. The specific absorption rate (SAR) level of the proposed antenna in both bands is below the European standard threshold. In summary, this wearable antenna sensor design shows clear advantages in simultaneously achieving information and power transfer for WBAN:

(1) Two different operating modes in a single button topology are used for information and power transfer, respectively.

(2) In the higher band, circular polarization and high efficiency are the beneficiaries of energy harvesting applications.

(3) A wideband AMC structure reduces the profile of the button, enhancing the stability of the antenna sensor and mimicking a realistic situation.

The main contribution of this paper is that a wearable button antenna sensor dedicated to a novel dual-mode wireless information and power transfer implementation is realized, by combining AMC and crossed dipole structures.

This paper is organized as follows: Firstly, the system analysis of the novel dual-mode wireless information and power transfer implementation for WBAN is discussed in Section 2. Next, the detailed dual-mode antenna sensor design is introduced in Section 3, followed by the measurement and simulation results in Section 4. Finally, concluding remarks are provided in Section 5.

## 2. System Analysis

**Figure 1:**
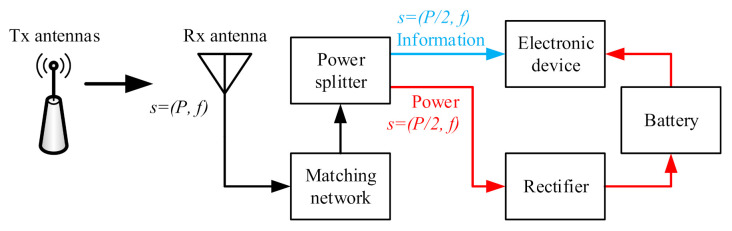
Typical SWIPT implementation for a single frequency.

A typical SWIPT system is illustrated in Figure 1 [29]. This system consists of one or more Transmit antenna (Tx antenna) sources and one user terminal, which is capable of decoding information and harvesting wireless radio frequency (RF) power simultaneously. The signal received by the Receive antenna (Rx antenna) is split in the information part (indicated in blue in Figure 1) and the power part (indicated in red in Figure 1). The information signal is sent to the electronic device directly, while power is sent to the rectifier and then stored in the battery. Assume that the received signal *s* has power *P* and frequency *f*. The splitting of the signal reduces the signal levels in each path.

**Figure 2:**
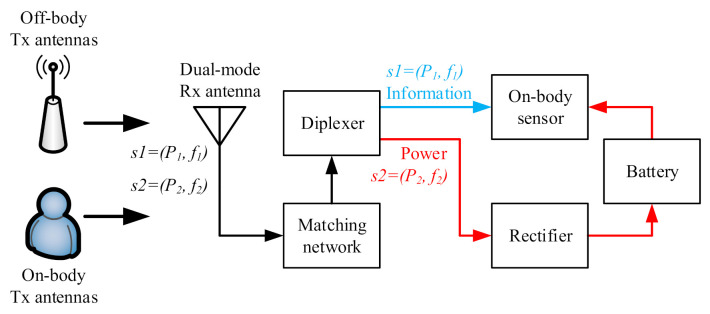
Proposed dual-mode wireless information and power transfer implementation involving two frequencies.

In this work, a novel system configuration is proposed, as shown in Figure 2, which is dedicated to minimizing this power loss. In the new scenario, the on-body Tx antennas communicate with the on-body sensors at frequency *f1*, while the off-body Tx antennas (e.g., the base station or the Wi-Fi antennas) serve as the power supply at frequency *f2*, respectively. The signals received by the dual-mode antenna are *s1* and *s2*, which are used for the information transfer and power harvesting, respectively. The frequency and power of *s1* and *s2* are (*f1*, *P1*) and (*f2*, *P2*), respectively. Theoretically, by using the diplexer the two signals at different frequencies can be separated without power loss.

The proposed configuration shows superior performance in terms of both thepower and information transmission efficiency. The input power entering the power path and the information path of a traditional system is lower than in the proposed novel system, since the splitting of the signal in the traditional system reduces the signal levels in each path to the average by 3 dB, without taking into account any other losses. For the power path, a smaller input power will typically lead to a lower power conversion efficiency (PCE) in the forward region of the diode [30]. For the information path, a smaller input power will reduce the signal-to-noise ratio (SNR), and therefore reduce the channel capacity [31].

In this way, the PCE for the energy harvesting path, and the channel capacity for the information transmission path, can be kept high compared with a traditional SWIPT system. The diplexer, which is commonly more complex than a power splitter, is used in the proposed design. A tradeoff has been made between the system complexity and its performance.

The proposed scenario is ideal for situations where off-body Tx antennas serving as power sources can be found, for example base stations or wireless fidelity (Wi-Fi) routers, operating at a different frequency than the frequency used for the information transfer. In many practical situations, this is indeed the case.

## 3. A Dual-Mode Wearable Button Antenna Sensor

**Figure 3:**
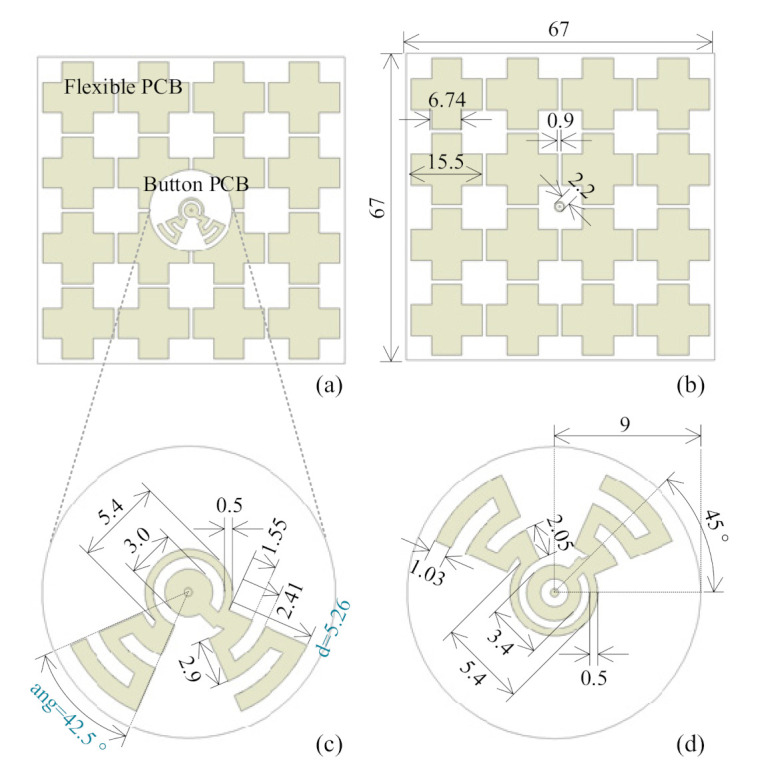
The topology of the proposed antenna sensor. (**a**) Top view of the proposed antenna sensor, (**b**) top view of the flexible PCB, (**c**) top view of the button PCB, and (**d**) bottom view of the button PCB. Dimensions are in mm.

**Figure 4:**
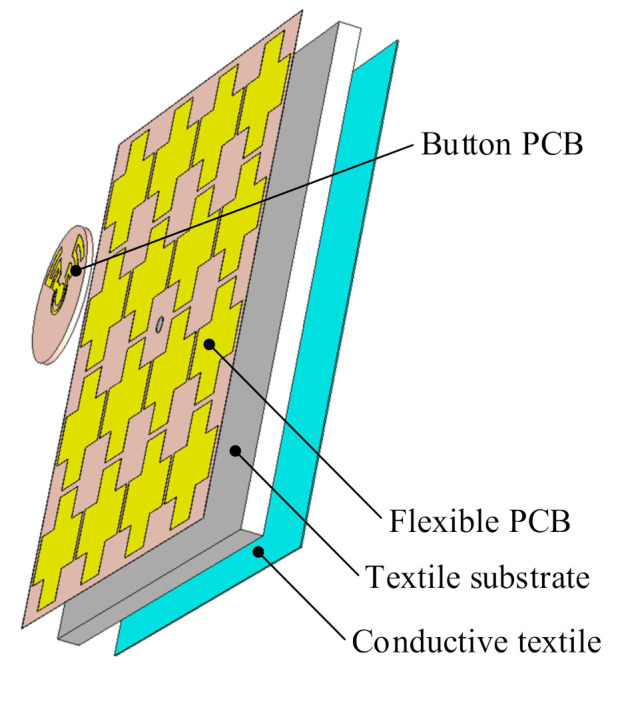
Exploded view of the proposed antenna sensor.

**Figure 5:**
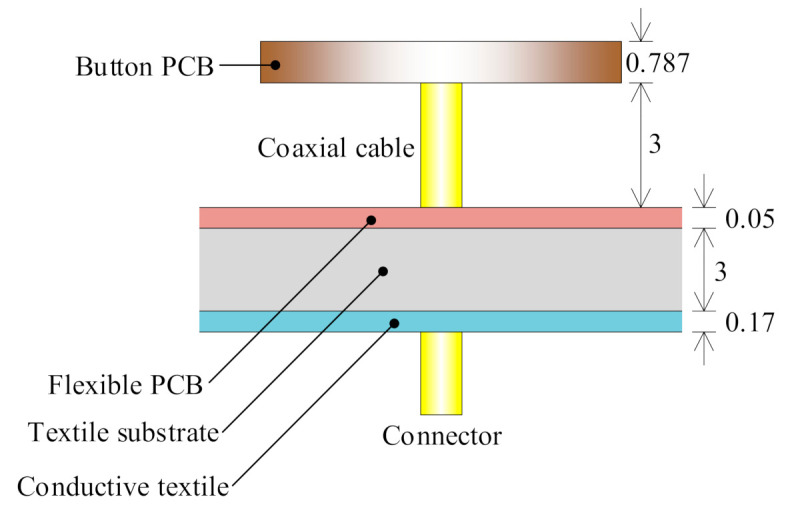
Side view of the proposed antenna sensor. Dimensions are in mm.

The proposed antenna is designed to operate in the 3.5 GHz WiMAX band, which is commonly used for on-body communication [32], and the 5 GHz WLAN band, which serves various wireless applications, indicating an advantage for wireless power harvesting. The topology of the proposed antenna sensor is given in Figure 3. The design was completed with CST Microwave Studio [33]. The radiator was conformally designed with a rigid button, which is combined with three flexible layers, as shown in the exploded view in Figure 4 and the side view in Figure 5. The rigid button printed circuit board (PCB) is made of RO4003C substrate (thickness 0.787 mm, relative permittivity 3.55, dielectric loss tangent 0.0027). The button PCB is compact and compatible with normal buttons, with a radius of only 9 mm. As we can see from Figure 3a,b, the rigid button PCB substrate carries the radiator structure on both sides. The flexible parts of the antenna are composed of a flexible PCB (thickness 0.05 mm, relative permittivity 4.3, dielectric loss tangent 0.025), a textile substrate (thickness 3 mm, relative permittivity 1.4, dielectric loss tangent 0.044) and a conductive textile (thickness 0.17 mm, conductivity of 118,000 S/m). The metal on the button PCB and the flexible PCB is copper, and the metal on the conductive textile is nickelate. In this proof-of-concept design, we chose to use a flexible PCB to carry the AMC layer to avoid the manual fabrication process of a complex conductive textile structure (AMC structure in this design), similar to that done in a previous design [34]. Note that, for future realistic design, the AMC may be made of the conductive textile. In this way, an AMC unit cell with simple topology is important for the fabrication process. A textile substrate is used to mimic cloth, and placed above the conductive textile, which acts as the reflector (ground). The flexible PCB layer is assembled to the textile substrate by using glue along the edge of the substrate. The conductive textile ground is directly adhered to the textile substrate by heating (using an electric iron). A coaxial cable is used to feed the antenna and support the button PCB.

The proposed antenna sensor is inspired by a crossed dipole antenna topology. As we can see from Figure 3, the designed dipole is meandered to achieve a compact design. A resonance at higher frequencies with wide bandwidth is achieved, and a circularly polarized broadside radiation pattern around 5.5 GHz is obtained by the crossed dipole structure. In addition, a monopole-like resonance with omni-directional radiation pattern is achieved at 3.5 GHz, which explicitly distinguishes this work from other crossed dipole antenna designs. As a result, two resonant modes are obtained. The lower resonance with monopole-like radiation pattern can support on-body communication. The higher operating band with broadside radiation pattern and circular polarization can be utilized for wireless power harvesting.

A coaxial cable is used to feed the antenna sensor. The outer conductor of the coaxial cable is soldered to the soldering pad of the bottom radiator. The inner conductor threads the button PCB and is soldered to the center of the radiator on the top layer.

**Figure 6:**
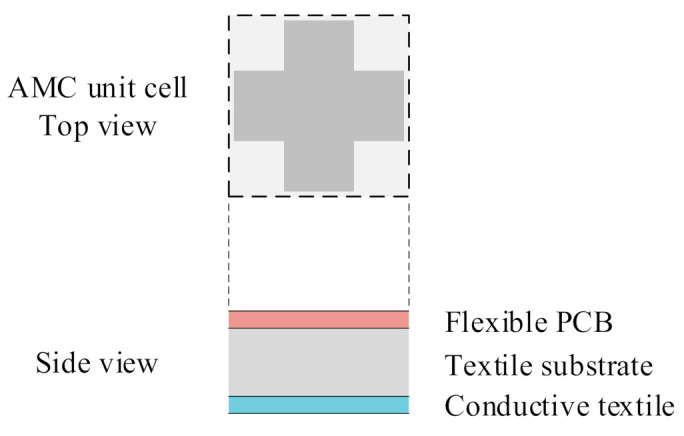
Top view and side view of the AMC structure.

**Figure 7:**
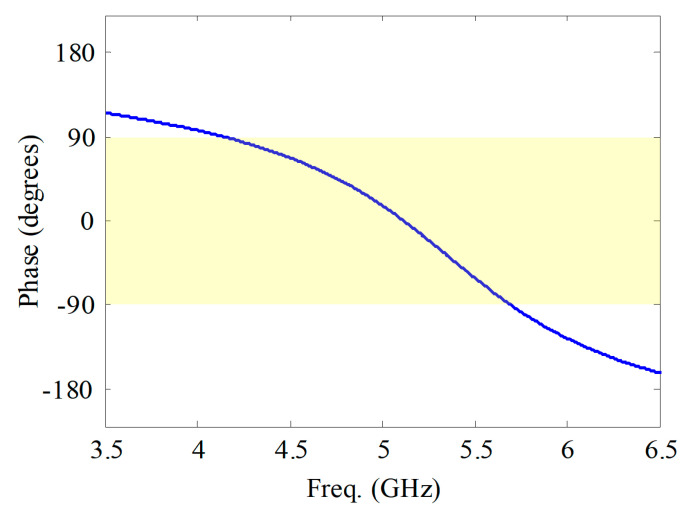
Phase of reflection coefficient of the AMC structure.

Traditional button antennas typically have a high profile, which may reduce the users’ comfort. The broadside radiation pattern in the higher band is obtained based on a dipole-with-reflector concept, which usually has a high profile of 0.25 wavelength (λ) at the resonant frequency. In wearable antenna sensor design, an AMC structure can be used to reduce the profile, and to isolate the user’s body from undesired electromagnetic radiation [35,36]. In our design, the spacing between the flexible layer (cloth) and the rigid PCB (button) is only 3 mm, thanks to the AMC structure shown in Figure 6. This value would be 13 mm without the AMC structure (the rest of the topology and the dimensions of the antenna being the same), which is very high. The supporting cable between the flexible layers and the rigid button is thus very short. This makes the designed antenna sensor more stable. The reflection coefficient (phase distribution) of the AMC structure with a plane wave incident from the normal direction is given in Figure 7. A perfect magnetic conductor (PMC)-like performance (reflected phase is 0°) is obtained in the targeted higher band. The operating bandwidth of the AMC structure can be defined by the phase of the reflected wave lying between −90° and 90°. So, the bandwidth is 4.1–5.7 GHz, which is wide enough, since this AMC structure is designed for the broadside radiation mode in the higher 5 GHz band. Electromagnetic simulations indicate that the omni-directional radiation at the 3.5 GHz band is excited with negligible effect of the AMC structure. Therefore, by introducing the AMC structure, the height of the antenna sensor is successfully reduced.

## 4. Prototype Performance

**Figure 8:**
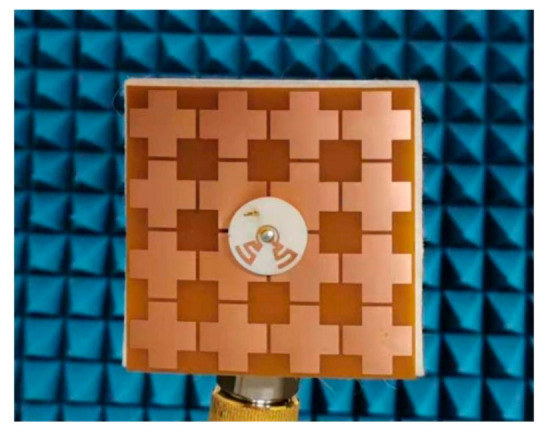
The prototype of the proposed antenna sensor.

**Figure 9:**
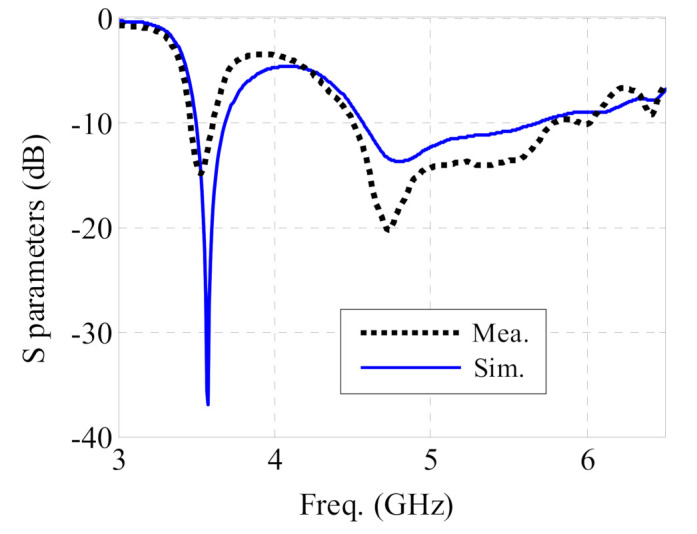
S parameter of the proposed antenna sensor.

The proposed antenna sensor was prototyped, see Figure 8. The S-parameters were measured with an HP 8520 Vector Network Analyzer. The simulated and measured S-parameters are presented in Figure 9. Dual-band operation is clearly observed. The measured −10 dB return loss bandwidths are 4.0% (3.47–3.61 GHz) in the lower band, and 25.0% (4.51–5.80 GHz) in the higher band, respectively. As a result, the proposed antenna fully covers the 3.5–3.6 GHz WiMAX band and the 4.6–5.8 GHz WLAN band. The simulated −10 dB bandwidths are 5.3% (3.50–3.69 GHz), and 21.8% (4.57–5.69 GHz). The discrepancy between simulations and measurements is mainly due to the manual fabrication procedure for the button PCB. The differences mainly occur at an absolute level of −10 dB and below. Considering the benchmarking study made in [37], this can be seen as acceptable.

**Figure 10:**
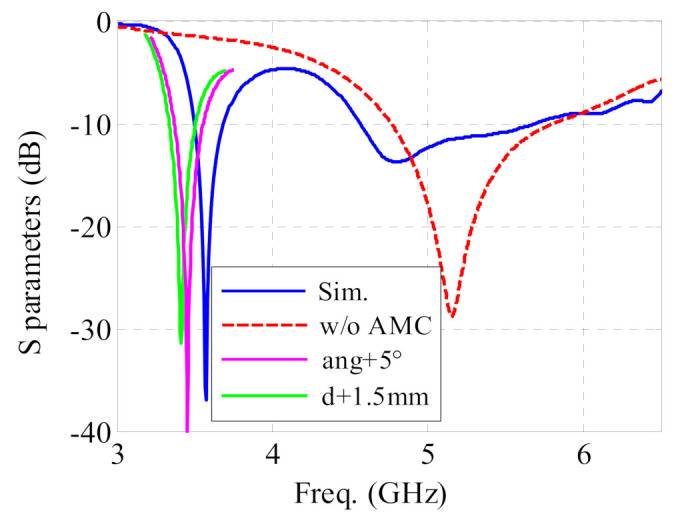
S parameter of the proposed antenna sensor. The red line indicates the result of the antenna without the AMC structure.

The simulated S-parameters of the antenna sensor without the AMC structure are indicated in the red line of Figure 10. Only one resonance is observed in this case. The antenna sensor performance is obviously determined by the antenna dimensions. Only the most important trends will be discussed here. The lower resonance is mainly affected by the flare angle “ang” of the sector structure and the length “d” of the crossed dipole, as shown in Figure 3. A larger angle and a longer length move the lower resonance to lower frequencies, as shown in Figure 10. The situation for the higher resonance is much more complicated. There, the impedance matching depends on multiple parameters. Only by optimizing the complete topology can the higher resonance, including matching, be tuned to other frequencies. Based on the aforementioned mechanism, the proposed design can be rescaled to other frequencies, and can be redesigned to other applications.

**Figure 11:**
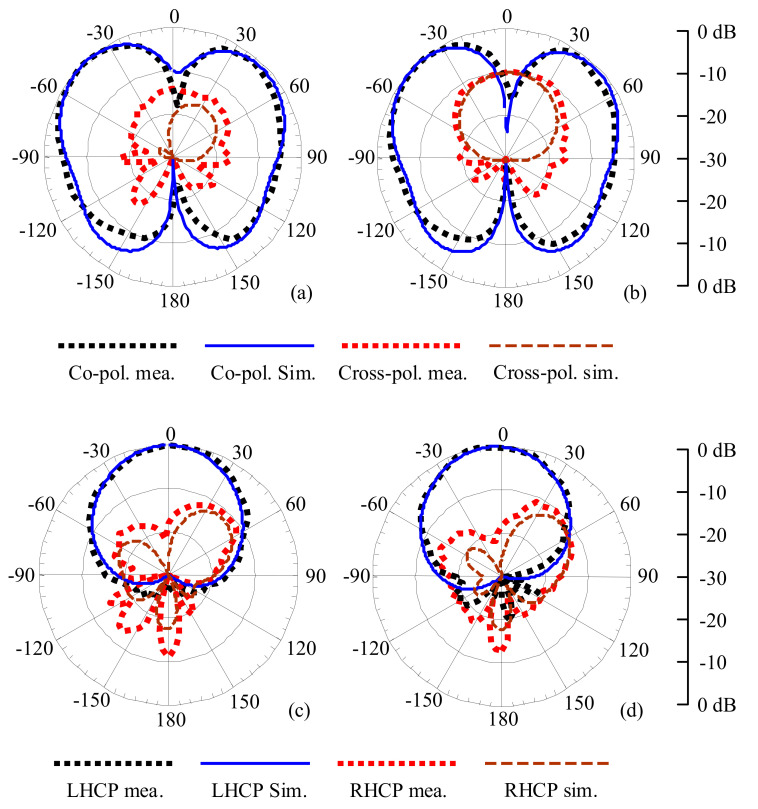
Normalized radiation pattern. (**a**) E-plane at 3.55 GHz. (**b**) H-plane at 3.55 GHz. (**c**) E-plane at 5.5 GHz. (**d**) H-plane at 5.5 GHz.

The radiation patterns were measured in the anechoic chamber at KU Leuven. Figure 11 gives the measured and simulated normalized radiation patterns of the proposed antenna. A representative monopole-like omni-directional pattern in the 3.5 GHz band and left-handed circularly polarization (LHCP) patch-like pattern in the 5 GHz band are observed. The cross-polarization level is below −10 dB for all patterns. The realized gain and total efficiency of the proposed antenna are summarized in Table 1.

**Figure 12:**
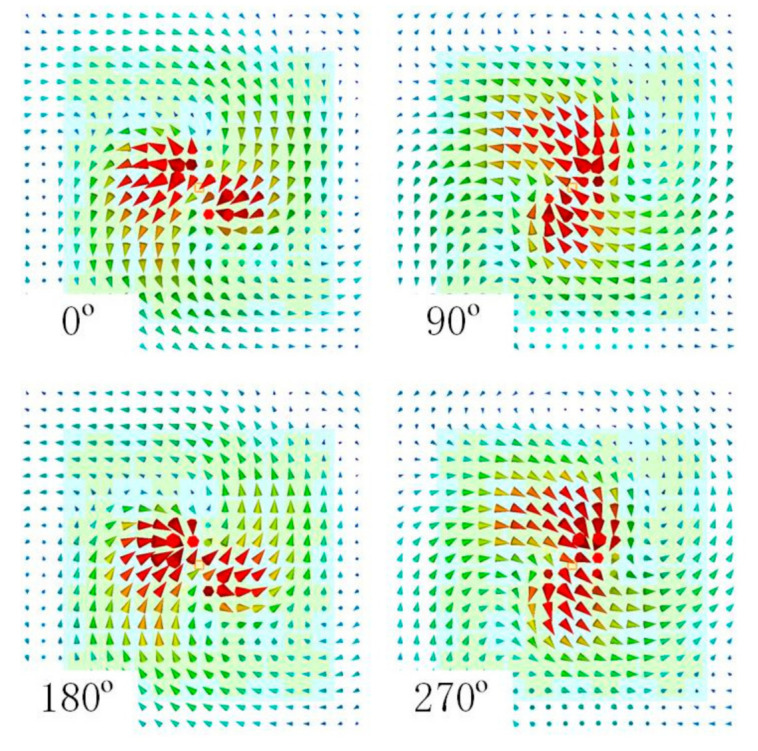
Simulated E-field 10 mm above the antenna ground.

**Figure 13:**
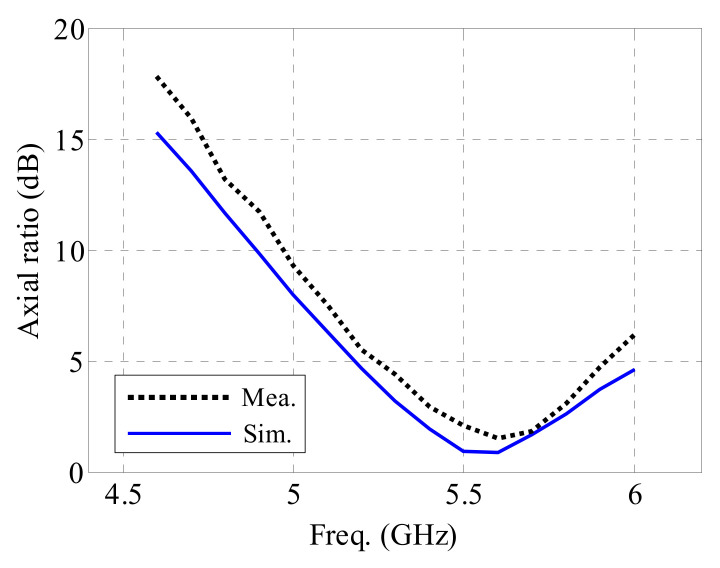
AR values in the *z*-axis direction in the higher band.

**Figure 14:**
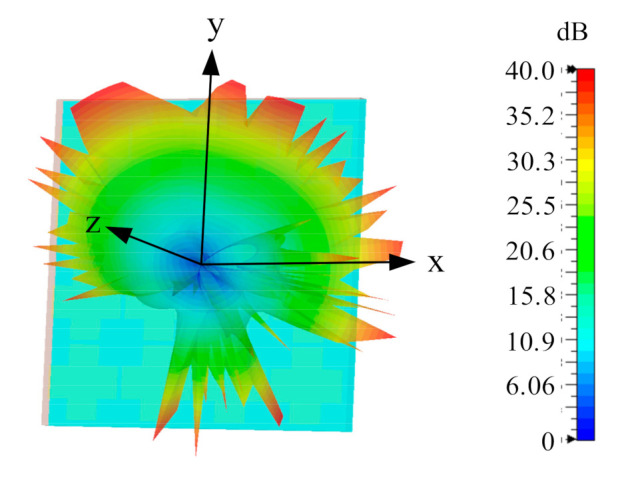
AR pattern at 5.5 GHz.

The axial ratio (AR) characteristic is crucial for circularly polarized radiation patterns. LHCP is achieved in this design. Figure 12 shows the simulated electrical field (E-field) 10 mm above the antenna ground at four different phases. In Figure 13, the AR results are shown. The measured 3 dB AR bandwidth is 7.0% (5.39–5.78 GHz), while the simulated bandwidth is 9.1% (5.32–5.83 GHz). The difference between measurements and simulations may come from the unavoidable fabrication tolerances and misalignments. The AR pattern at 5.5 GHz can be seen in Figure 14. The AR values around the *z*-axis (radiation direction) are quite good. At 5.5 GHz, the 3 dB AR angular spans in both the xz and yz planes are 35°. 

**Figure 15:**
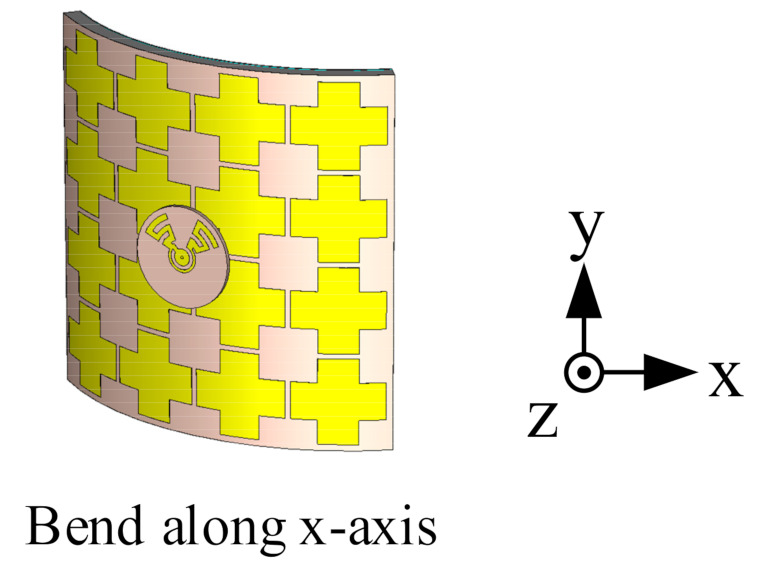
Bending over a cylinder along the *x*-axis.

**Figure 16:**
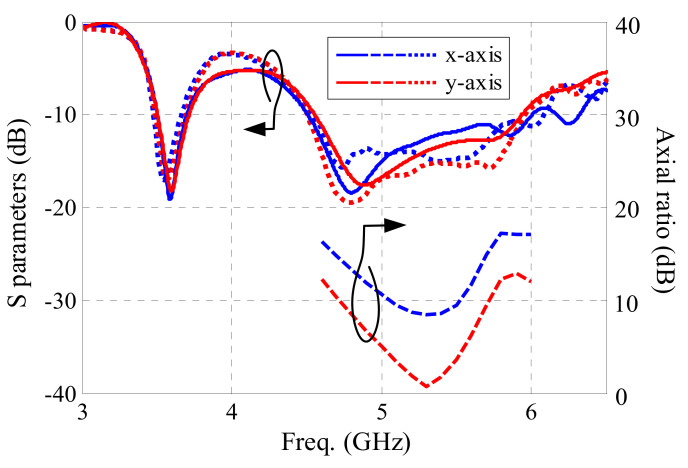
Antenna sensor performance under bending. The blue lines indicate the bending case along *x*-axis. The red lines indicate the bending case along *y*-axis. The solid lines indicate the measurement results. The dashed and dotted lines indicate the simulation results.

In order to research the impact of actual wearable situations on the antenna performance, bending and tilting cases were considered. Firstly, the flexible parts (flexible PCB, textile substrate, and conductive textile) of the antenna sensor were bent over a cylinder along the *x*- and *y*-axis with a radius of 50 mm, as shown along the *x*-axis in Figure 15. The antenna performance is shown in Figure 16. Both measured and simulated −10 dB bands cover 3.50–3.65 GHz for the lower band, and 4.56–5.93 GHz for the higher band, which indicates that the reflection coefficient of the antenna is not significantly affected by bending. The AR result for *x*-axis bending is worse, while the one for *y*-axis bending moves to a lower frequency. The 3 dB AR band for *y*-axis bending is 5.13–5.47 GHz.

**Figure 17:**
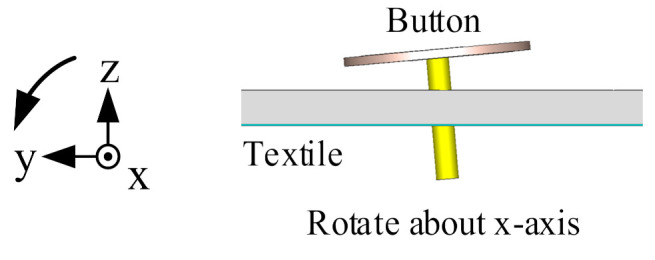
Rotating about the *x*-axis.

**Figure 18:**
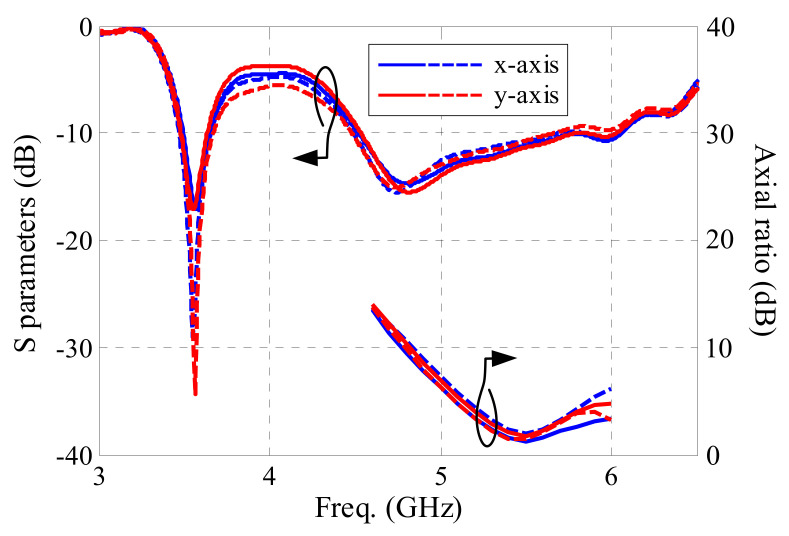
Antenna sensor performance when tilting. The blue lines indicate tilting about the *x*-axis. The red lines indicate tilting about the *y*-axis. The solid lines indicate a rotation angle of −5°. The dashed lines indicate a rotation angle of 5°.

Secondly, the button PCB, along with the supporting coaxial cable, was rotated about the *x*- and *y*-axes with different angles, as shown along the *x*-axis in Figure 17. The simulated reflection coefficient and AR performance are shown in Figure 18. As seen from Figure 18, the lower and higher bands for all cases are not affected significantly, covering 3.50–3.63 GHz and 4.54–5.65 GHz for the lower and higher band, respectively. The AR results for all tilting cases are also acceptable. The 3 dB AR band of the worst given case is 5.32–5.68 GHz.

**Figure 19:**
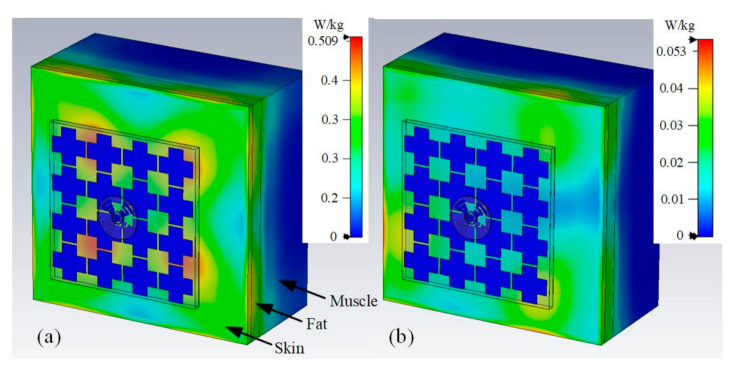
SAR of the proposed antenna (**a**) at 3.55 GHz, and (**b**) at 5.5 GHz.

In order to ensure conformance to safety regulations, the SAR of the proposed antenna was simulated using a simplified human chest model (see Figure 19). This model was configured to be 10 mm behind the antenna sensor to mimic the practical antenna-to-skin distances due to clothing. The model consists of a skin layer, a fat layer, and a muscle layer, with the thicknesses 3 mm, 7 mm, and 40 mm, respectively. The input power to the antenna was set at 0.5 W (rms). The SAR values were calculated based on the IEEE C95.1 standard [38] and averaged over 10 g of biological tissue. The results shown in Figure 19 indicate that the SAR is below 0.509 W/kg at 3.55 GHz, and below 0.0532 W/kg at 5.5 GHz, respectively, which is much lower than the European standard threshold of 2 W/kg.

Table 2 gives a comparison of wearable antennas for wireless power transfer in literature. The superiority of the proposed antenna sensor is clearly shown. The designed antenna sensor reaches excellent combined performances, e.g., a wide bandwidth, circularly polarized radiation, a very small radiator size, and high efficiency.

## 5. Conclusions

A novel dual-mode wearable button antenna sensor is proposed. With this antenna sensor, a new dual-mode wireless information and power transfer implementation for WBAN was realized, where two transmission paths at two different frequencies are utilized for the information and power transfer, respectively, yielding an increased PCE and channel capacity. The lower resonance is in the 3.5 GHz band (3.47–3.61 GHz), and has a monopole-like pattern, which is suitable for on-body information transfer. The higher resonance is in the 5 GHz band (4.51–5.80 GHz) and has a patch-like pattern with circular polarization, which is suitable for off-body wireless power harvesting. A wideband flexible AMC structure was introduced to realize a low-profile design (0.08 λ at 3.5 GHz). The proposed design integrates two operating modes with an unprecedented combination of properties, such as low profile, flexibility, high efficiency, and wideband, which enables a new application scenario of simultaneous wireless information and power transfer.

## Figures and Tables

**Table 1:** Realized gain and radiation efficiency.

Freq. (GHz)	Realized Gain		Radiation Efficiency	
	Sim.	Mea.	Sim.	Mea.
3.55 GHz	2.7 dBi	2.9 dBi	87%	90%
5.0 GHz	7.9 dBi	8.4 dBi	83%	90%
5.5 GHz	7.2 dBi	7.9 dBi	81%	87%

The realized gain values indicate the peak gains.

**Table 2:** Comparison of wearable antennas for wireless power transfer.

Ref.	Freq. Band		Dual-Band	Circularly Polarized	Efficiency (Sim.) in %	Radiator Size in λ^2^	Antenna Size in λ^2^	Bending Analysis	Tilting Analysis
	in GHz	Mea.							
[15]	2.45	N/A	No	No	70	0.36 × 0.41	0.44 × 0.49	Yes	No
[18]	1.94–3.03	43.7	No	Yes	55	0.49 × 0.49	0.49 × 0.49	Yes	No
[19]	2.45/5.40–6.15	4/12	Yes	No	N/A	0.44 × 0.44	0.98 × 0.98	Yes	No
[22]	ca. 2.36–2.72	14.2	No	No	75	N/A	0.73 × 0.98	Yes	No
[23]	ca. 2.43–2.48	2.0	No	No	89	0.44 × 0.30	0.55 × 0.57	No	No
[24]	2.23–2.66/4.62–5.07	17.6/9.3	Yes	No	N/A	0.34 × 0.36	0.36 × 0.41	Yes	No
[25]	2.47–2.60	5.2	No	Yes	N/A	0.17 × 0.17	0.29 × 0.29	No	No
[26]	2.39–2.57/4.80–8.18	7.3/52.1	Yes	Yes	N/A	0.15 × 0.15	0.51 × 0.51	Yes	Yes
This work	3.47–3.61/4.51–5.80	4.0/25.0	Yes	Yes	90 / 87	0.21 × 0.21	0.78 × 0.78	Yes	Yes

λ indicates the wavelength in free space of the lowest operating frequency. The comparison of efficiency is considered when the antenna is in free space.

## Data Availability

Not applicable.

## References

[1] Ayed S., Chaari L., Fares A. (2020). A survey on trust management for WBAN: Investigations and future directions. Sensors.

[2] Simorangkir R.B.V.B., Kiourti A., Esselle K.P. (2018). UWB wearable antenna with a full ground plane based on PDMS-embedded conductive fabric. IEEE Antennas Wirel. Propag. Lett..

[3] Zhang J., Yan S., Hu X., Vandenbosch G.A.E. (2020). Mutual coupling suppression for on-body multi-antenna systems. IEEE Trans. Electromagn. Compat..

[4] Abbasi Q.H., Ur-Rehman M., Qaraqe K., Alomainy A. (2016). Advances in Body-Centric Wireless Communication: Applications and State-of-the-Art.

[5] Alqadami A.S., Bialkowski K.S., Mobashsher A.T., Abbosh A.M. (2018). Wearable electromagnetic head imaging system using flexible wideband antenna array based on polymer technology for brain stroke diagnosis. IEEE Trans. Biomed. Circuits Syst..

[6] Memon A.W., Paula I.L., Malengler B., Vasile S., Torre P.V., Langenhove L.V. (2021). Breathable textile rectangular ring microstrip antenna at 2.45 GHz for wearable applications. Sensors.

[7] Elias B.B.Q., Soh P.J., Al-Hadi A.A., Akkaraekthalin P., Vandenbosch G.A.E. (2021). Bandwidth optimization of a textile PIFA with DGS using characteristic mode analysis. Sensors.

[8] Khan M.M., Rahman M.A., Talha M.A., Mithila T. (2014). Wearable antenna for power efficient on-body and off-body communications. J. Electromagn. Anal. Appl..

[9] Azaro R., Debiasi L., Zeni E., Benedetti M., Rocca P., Massa A. (2009). A hybrid prefractal three-band antenna for multistandard mobile wireless applications. IEEE Antennas Wirel. Propag. Lett..

[10] Li S., Zhou X., Wang C.-X., Yuan D., Zhang W. (2017). Joint transmit power allocation and splitting for SWIPT aided OFDM-IDMA in wireless sensor networks. Sensors.

[11] Chen H., Li Y., Jiang Y., Ma Y., Vucetic B. (2015). Distributed power splitting for SWIPT in relay interference channels using game theory. IEEE Trans. Wirel. Commun..

[12] Shadid R., Haerinia M., Roy S., Noghanian S. (2018). Hybrid inductive power transfer and wireless antenna system for biomedical implanted devices. Prog. Electromagn. Res. C.

[13] Zhang J., Yan S., Vandenbosch G.A.E. (2018). Realization of dual band pattern diversity with a CRLH-TL inspired reconfigurable metamaterial. IEEE Trans. Antennas Propag..

[14] Hu X., Yan S., Vandenbosch G.A.E. (2017). Wearable button antenna for dual-band WLAN applications with combined on and off-body radiation patterns. IEEE Trans. Antennas Propag..

[15] Adami S.-E., Proynov P., Hilton G.S., Yang G., Zhang C., Zhu D., Li Y., Beeby S.P., Craddock I.J., Stark B.H. (2018). A flexible 2.45-GHz power harvesting wristband with net system output from −24.3 dBm of RF power. IEEE Trans. Microw. Theory Tech..

[16] Shaw T., Samanta G., Mitra D., Mandal B., Augustine R. (2021). Design of metamaterial based efficient wireless power transfer system utilizing antenna topology for werable devices. Sensors.

[17] Zhang J., Meng J., Li W., Yan S., Vandenbosch G.A.E. A Metamaterial Inspired Button Antenna for Wireless Power and Data Transfer. Proceedings of the 2020 IEEE 3rd International Conference on Electronic Information and Communication Technology (ICEICT).

[18] Lui K.W., Murphy O.H., Toumazou C. (2013). A wearable wideband circularly polarized textile antenna for effective power transmission on a wirelessly-powered sensor platform. IEEE Trans. Antennas Propag..

[19] Zhu S., Langley R. (2009). Dual-Band Wearable Textile Antenna on an EBG Substrate. IEEE Trans. Antennas Propag..

[20] Zhang X.Y., Wong H., Mo T., Cao Y.F. (2017). Dual-band dual-mode button antenna for on-body and off-body communications. IEEE Trans. Biomed. Circuits Syst..

[21] Sanz-Izquierdo B., Huang F., Batchelor J.C. (2006). Covert dual-band wearable button antenna. Electron. Lett..

[22] Lemey S., Declercq F., Rogier H. (2014). Dual-band substrate integrated waveguide textile antenna with integrated solar harvester. IEEE Antennas Wirel. Propag. Lett..

[23] Vital D., Bhardwaj S., Volakis J.L. (2020). Textile-based large area RF-power harvesting system for wearable applications. IEEE Trans. Antennas Propag..

[24] Zhang W., Zhuang Y., Song C., Huang Y., Zhou J. A dual-band quasi-Yagi wearable antenna with high directivity. Proceedings of the 2018 IEEE MTT-S International Wireless Symposium (IWS).

[25] Agarwal K., Nasimuddin, Alphones A. (2013). RIS-based compact circularly polarized microstrip antennas. IEEE Trans. Antennas Propag..

[26] Yin X., Chen S.J., Fumeaux C. (2020). Wearable Dual-Band Dual-Polarization Button Antenna for WBAN Applications. IEEE Antennas Wirel. Propag. Lett..

[27] Wen L.-H., Gao S., Luo Q., Mao C.-X., Hu W., Yin Y., Zhou Y., Wang Q. (2018). Compact Dual-Polarized Shared-Dipole Antennas for Base Station Applications. IEEE Trans. Antennas Propag..

[28] Tran H.H., Park I., Nguyen T.K. (2017). Circularly polarized bandwidth-enhanced crossed dipole antenna with a simple single parasitic element. IEEE Antennas Wirel. Propag. Lett..

[29] Pan N., Rajabi M., Claessens S., Schreurs D., Pollin S. (2020). Transmission strategy for Simultaneous wireless information and power transfer with a non-linear rectifier model. Electronics.

[30] Hu Y.Y., Sun S., Xu H., Sun H. (2019). Grid-array rectenna with wide angle coverage for effectively harvesting RF Energy of low power desity. IEEE Trans. Microw. Theory Tech..

[31] Chen C.M., Volski V., Van der Perre L., Vandenbosch G.A.E., Pollin S. (2017). Finite large antenna arrays for Massive MIMO: Characterization and system impact. IEEE Trans. Antennas Propag..

[32] Utsav A., Kumar A., Badhai R.K., Suraj P. A dual band on body monopole antenna for WiMAX and X-band applications. Proceedings of the 2018 International Conference for Convergence in Technology (I2CT).

[33] Microwave Studio (2016). Computer Simulation Technology (CST). http://www.cst.com/Products/CSTMWS/.

[34] Yan S., Vandenbosch G.A.E. (2016). Radiation pattern-reconfigruable wearable antenna based on metamaterial structure. IEEE Antennas Wirel. Propag. Lett..

[35] Agarwal K., Guo Y.-X., Salam B. (2016). Wearable AMC backed near-endfire antenna for on-body communications on latex substrate. IEEE Trans. Compon. Packag. Manuf. Technol..

[36] Raad H.R., Abbosh A.I., Al-Rizzo H.M., Rucker D.G. (2013). Flexible and compact AMC based antenna for telemedicine applications. IEEE Trans. Antennas Propag..

[37] Vandenbosch G.A.E., Mioc F., Saporetti M., Foged L. (2016). Bridging the simulations—measurements gap: State-of-the-art. IEEE Antennas Propag. Mag..

[38] (2019). IEEE.IEEE C95.1-2019. http://standards.ieee.org/standard/C95_1-2019.html.

